# Prevalence and associated factors of depression among Jimma University students. A cross-sectional study

**DOI:** 10.1186/s13033-020-00384-5

**Published:** 2020-07-28

**Authors:** Gutema Ahmed, Alemayehu Negash, Habtamu Kerebih, Daniel Alemu, Yonas Tesfaye

**Affiliations:** 1 Department of Psychiatry, Faculty of Medicine, Jimma University, Jimma, Ethiopia; 2 Department of Psychiatry, University of Gondar, Gondar, Ethiopia; 3 Department of Psychiatry, Haramaya University, Harar, Ethiopia

**Keywords:** Depression, Jimma University, Emotional distress, Academic performance

## Abstract

**Background:**

Depression is a common health problem among university students. It is debilitating and has a detrimental impact on students psychosocial, emotional, interpersonal functioning and academic performance, However, there is a scarcity of information on this regard in higher education institutions in Ethiopia, so the current study was conducted to assess the prevalence of depression and its associated factors among Jimma University students.

**Methods:**

An institution-based quantitative cross-sectional study was conducted on a total of 556 sampled students selected by a multistage stratified sampling technique. Beck Depression Inventory (BDI-II) was used to screen depression severity. Data was collected through a pretested, structured, and self-administered questionnaire. The collected data were checked manually for completeness and entered into Epidata manager Version 2.0.8.56 data entry software then exported to SPSS version 20 Statistical software for analysis. The obtained data were described using descriptive statistics as well as logistic regression analysis was done to determine the independent predictors of the outcome variable. First bivariate analysis was done and variables significant at *p* value ≤ 0.25 were entered into a multivariate logistic regression analysis to control for confounders. The significance of association was determined at a 95% confidence interval and p-value < 0.05.

**Result:**

The prevalence of depression among the students was 28.2%. Having a mentally ill family member (OR = 2.307, 95%CI 1.055–5.049), being from the college of Social science and humanity (OR = 2.582, 95%CI 1.332–5.008), having sex after drinking (OR = 3.722, 95%CI 1.818–7.619), being hit by sexual partner (OR = 3.132, 95%CI 1.561–6.283), having childhood emotional abuse (OR = 2.167, 95%CI 1.169–4.017), having monthly pocket money between 500-999 ETB (OR = 0.450, 95% CI 0.204–0.995), and promoted academic performance (OR = 2.912, 95% CI 1.063–7.975) were significantly associated with depression.

**Conclusion:**

The prevalence of depression among Jimma University students was high and positively associated with being from the college of social science and humanity, history of a hit by a sexual partner, having a mentally ill family member, having more monthly pocket money, promoted academic performance, having sex after drinking and childhood emotional abuse. Therefore, establishing depression screening services on the campus and designing proper mental health intervention programs is recommended to tackle the problem.

## Background of the study

Depression is a common and severe mental disorder [1, 2] caused by the combinations of genetic [2, 3], social, psychological and environmental factors [1, 3]. Globally the prevalence of depression is increasing at an alarming rate [2, 3]. Depressive disorders are the most pressing public health problems associated with substantial impairment, comorbidity, poor quality of life, and high mortality [1, 3]. It affects economic activity [3, 4], learning, social [5, 6], cultural life [3, 7] and people’s ability to participate in their communities [2, 4]. Depression also poses a risk of suicide and suicide attempts [1, 2, 4, 8, 9] in young adults.

University students are a special segment of the society at a specific developmental milestone that bridges a critical transitory period from adolescence to adulthood [10]. This transitional period embraces a very important process like endocrine surge, emotional turmoil, and identity development which can lead to crises, which include self-doubt, social withdrawal, loneliness, lowered self-esteem [10–12]. University students are challenged by geographic changes [10], separated from family members [6, 10, 13], academic stress [10, 13–16], alongside financial difficulties urging the student to develop new coping mechanisms [8, 11, 17]. Therefore, University students worldwide are at higher risk to develop mental health disorders particularly to depression [14, 18], and suffer from its impact [19].

Various studies across the globe showed the prevalence of depression among university students varied, as low as 4% [20] and as high as 79.2% [21]. A systematic review of 35 studies conducted among university students in Iran from 1995 to 2012 showed that the prevalence of depression was 33% [22]. Different American countries had the prevalence of depression range between 33% and 41% [6, 23, 24]. The report on different European countries stated that the prevalence of depression ranges between 6.1% and 34.2% [5, 8, 25]. The study done in different Asian countries reported that the prevalence of depression ranges between 4% and 79.2% [14, 18, 20, 22, 26–36]. The study conducted in African Universities showed that the prevalence of depression ranges between 16.2% and 67% [13, 36–41]. In Ethiopia, the prevalence of depression among students is between 29.1% and 32.2% [9, 42].

Depression among university students is significantly associated positively and negatively with marital status [22], sex and gender [11–13, 23, 26, 32, 38, 42, 43], age [13, 20, 25, 33, 38], family problems [5, 7, 13, 14, 20, 25, 29, 37], parental education [5, 13, 20], social support [25], family history of mental illness [5, 14, 40], financial struggles [11, 17, 20, 25, 29, 38], academic achievement [6, 13], field of study [44], year of study [20, 25, 29, 32, 36, 40, 42], type of college [13], satisfaction of major study [5, 20, 25, 32], substance use (alcohol, tobacco and khat) [13, 14, 29, 37, 39, 40, 42, 45], risky sexual behaviour [37, 39], physical abuse [39], child abuse [39, 40], forced sexual experience [37, 39], sexual partner abuse [37] and background residence [8, 46]. As a result of depression, students missed greater number of classes, assignments, exams and even forced to dropout from the University [4, 6, 13, 15].

Persistent ignorance and misperceptions of the disease lead to painful stigmatization and avoidance of the diagnosis by many of those affected [33]. Having this high prevalence of depression among students; help and treatment-seeking behavior is very low [47, 48]. Therefore establishing proper screening and intervention programs [18, 29, 49] and educating the students through stress management programs and counseling them in the University is recommended for the overall reduction of mental illness [14, 32, 36, 50].

Social support has been shown to promote mental health and acts as a buffer against stressful life events [51]. A lack of social support is a determinant of mental health problems including depressive symptoms among university students [52, 53]. Research evidence indicates a significant negative relationship between social support and psychological disorders including depression and stress [52, 54, 55].

There is a scarcity of studies on the prevalence of depression and its predictors in the Ethiopian university students, moreover, depression was reported merged with other disorders and, limited variables were addressed in the previous studies. Therefore, studying depression on university students is vital to investigate the problem thoroughly and come up with important recommendations. Furthermore, the findings of this study will be fundamental for the University administrators and other stakeholders to screen and provide mental health services on the campus and further help interested researchers in the topic area to conduct additional studies on different study designs.

## Method and materials

### Study setting, design, population, and sampling technique

The study was conducted in Jimma University main campus from April 5 to 20, 2016. An institution-based quantitative cross-sectional study was conducted among 556 regular undergraduate main campus students of Jimma University. A multistage stratified sampling technique was used to select the study participants. All colleges in Jimma University’s main campus were included and stratification was done on the department level and year of the study. A total of four colleges and 30 departments were found on the main campus. Eleven departments were selected randomly by the lottery method. Each respondent was selected by simple random sampling technique using the enrollment registry as a frame. A total of 6155 regular undergraduate students found on the main campus in 2016.

### Sample size estimation

The sample size was estimated using a single population proportion formula; $\text{n = }\,\left( {{z\alpha }\,\text{ / }\,\text{2}} \right)^{\text{2}} \,\text{P}\,\left( {{1 - p}} \right)\,\text{ / }\,\text{d}^{\text{2}}$ considering, n; Sample size, z; critical value 1.96, α/2; confidence level, P; prevalence of depression at Hawassa college students = 23.6% [56], d; margin of error = 0.05 (5%). Accordingly, it becomes 277. Since the total population was less than ten thousand finite population correction formula was used to get the desired sample size, $\text{nf}\,\text{ = }\,\text{n}\,\text{ / }\,\left( {\text{1}\,\text{ + }\,\text{n}\,\text{ / }\,\text{N}} \right)$; where nf is the final sample size, n; the calculated sample size (277) and N; the total population(6155), hence it becomes 266. Since the sampling was multistage, the calculated sample size was multiplied by two for design effect and a 10% non-response rate was added to get the final sample size of 586.

### Data collection procedure and tools

A self-administered structured questionnaire was used to collect the data. The Questionnaire consisted of socio-demographic, economic, social, and environmental variables. The tool was developed after an extensive review of the literature on the topic area. Beck Depression Inventory (BDI-II) was used to screen the presence and the severity of depressive symptoms. The 21-item was scored on a scale of 0–3 in a list of four statements arranged in increasing severity about a particular symptom of depression. The total score ranges from 0 to 63. BDI scores of 14 or higher were categorized as the presence of depression for logistic regression analysis [20]. According to BDI-II: a score of 0 to 4 is (Normal), 5 to 13 is (Borderline clinical depression), 14 to 19 is (Mild depression), 20 to 28 is (Moderate depression), and 29 to 63 is (Severe depression) [7, 13, 20]. The internal consistency (Cronbach’s α) of the Beck Depression Inventory was ranging from 0.75–0.88 across different studies [11, 20, 35, 57], in this study the internal consistency was high (Cronbach’s α = 0.897). The level of Social support of the respondents was measured by Oslo 3-items social support scale. The tool was validated and has been used in previous studies in Ethiopia [58–60]. In this study, the tool had Cronbach’s α score of 0.91. A score of 3–8 indicates poor support, 9–11 is moderate support and 12–14 is strong support. Substance use was measured by the current user (A person who used any of the substances at least once in the past 30 days) and lifetime user (use of any of the substances at least once in an individual’s lifetime) [61].

The questionnaire was translated to the local languages (Amharic and Afaan Oromo) and back-translated to English to check its consistency, additionally, consensus version was developed in group discussions by involving different research experts of the field, this was compared with the original version and confirmed to be good for use by consultant mental health experts. Moreover, the face validity test was performed by three independent experts in the field. The questionnaire was pre-tested on 28 students to check the impending problems of the actual data collection. Finally, the Amharic and Afan Oromo version of the questionnaire was used to collect the data. The self-administered questionnaire was later collected from the respondents by the data collection facilitator after checking the completeness of the information.

### Data processing and analysis

The collected data were cleaned, coded, and entered into Epidata manager Version 2.0.8.56 data entry software and analyzed using SPSS version 20 data analysis software. Descriptive statistics (mean, percentage, frequencies, and standard deviation) were done to summarize the dependent and independent variables. Bivariate logistic regression analysis was done to see the association of each independent variable with the outcome variable. Accordingly, variables with p-value ≤ 0.25 were entered into a multivariate logistic regression to control the confounder. Finally, a p-value of less than 0.05 was considered statistically significant association, and the adjusted odds ratio with 95% CI was calculated to determine the strength of association.

### Data quality management

Regular supervision was made by the data collection supervisor to ensure that all necessary data were properly collected. Each day of data collection, the filled questioners were cheeked manually first for completeness and consistency then the collected data were processed timely and enter from a paper onto the computer twice. When incomplete questionnaires found the data collection supervisors kindly asked the respondents to fill the missed information, if the respondents were not willing to fill the missed information, the questionnaire was discarded from the analysis for gross incompleteness.

### Ethical approval and consent to participate

The study protocol was approved by the Institutional Review Board of Jimma University, Institute of Health. Written informed consent was obtained from each study participant. The involvement of the study participants was voluntarily and participants were informed of the right to withdraw anytime from the study. The data collection was undertaken confidentially and responses were kept private and anonymous. The study was conducted as per the Helsinki declaration.

## Result

### Socio-demographic, economic, academic and health status characteristics of the respondents

A total of 556 respondents participated in the study giving a response rate of 94.9%. From the total respondents, the majority were male 64.7% (n = 360). The mean age of the respondents was 21.21(SD = ± 1.99 years) with minimum and maximum age to be 18 and 35 years, respectively. Majority of the participants were Oromo, Orthodox Christians and single, 59% (n = 328), 37.8% (n = 210) and 86.0% (n = 478) respectively. Most of the participants 43.2%(n = 240) were from the College of Health Sciences and 32.7% (n = 182) were first-year students. The majority of 39.4% (n = 219) respondents earned monthly pocket money between 300–499 ETB. About 42.4% (n = 236) of the respondent has moderate social support. Among the respondents, 59.2% (n = 329) and 51.3% (n = 285) of students had academic work overload and overburdened by test schedules respectively. According to the participant’s response, 6.7% (n = 37) and 8.5% (n = 47) of the participant had a chronic physical illness and family members with mental illness respectively. Table 1.

**Table 1:** Socioeconomic and academic characteristics of participants among Jimma University main campus regular student, April 2016 (N = 556)

Variable	Frequency (n)	Percentage (%)
Gender		
Male	360	64.7
Female	196	35.3
Age		
18–20	231	41.5
21–22	212	38.1
23–35	113	20.3
Ethnicity		
Oromo	328	59
Amhara	130	23.4
Tigre	26	4.7
Gurage	32	5.8
Yem	14	2.5
Others*	26	4.7
Marital status		
Married	60	10.8
Single	478	86.0
Others**	18	3.2
Religion		
Muslim	182	32.7
Orthodox	210	37.8
Protestant	159	28.6
Others***	5	0.9
Childhood residence		
Rural	294	52.9
Urban	262	47.1
Monthly pocket money (Ethiopian Birr)		
100–299	107	19.2
300–499	219	39.4
500–999	186	33.5
1000–4000	44	7.9
Chronic physical illness		
No	519	93.3
Yes	37	6.7
Mother educational level		
Illiterate	176	31.7
Grade 1–8	232	41.7
Grade 9–12	111	20
University	37	6.7
Father educational level		
Illiterate	127	22.8
Grade 1–8	199	35.8
Grade 9–12	140	25.2
University	90	16.2
Family income (Ethiopian Birr)		
300–2000	205	36.9
2001–2999	121	21.8
3000–4999	151	27.2
5000–25,000	79	14.2
Parental relationship		
Good	322	57.9
Moderate/fair	135	24.3
Poor	99	17.8
Family history of mental illness		
No	509	91.5
Yes	47	8.5
Year of study		
1st year	182	32.7
2nd year	136	24.5
3rd year	122	21.9
4th year	63	11.3
5th year	53	9.5
College		
CHS	240	43.2
CSSH	125	22.5
CNCS	93	16.7
CLG	98	17.6
Academic performance		
Warned	4	0.7
Promoted	345	62.1
Distinction	167	30
Great distinction	39	7
Academic work overload		
No	227	40.8
Yes	329	59.2
Satisfaction with major study		
Good	295	53.1
Moderate/fair	162	29.1
Poor	99	17.8
Overburdened with the test schedule		
No	271	48.7
Yes	285	51.3
Social support		
Poor support	103	18.5
Moderate Support	236	42.4
Strong Support	217	39.0

*CHS* College of health science, *CSSH* College of social science and Humanity, *CNCS* College of natural and computational science, *CLG*College of law and Governance

*Somali, Afar and Wolayta, etc.

**Divorced, Widowed, and Separated

***Wakefata, Catholic and Athiest

### Substance use, risky sexual behavior, and negative life events characteristics of the respondents

This study revealed that 66% (n = 78) of the respondents currently chew khat and 24.2% (n = 51) were drinking alcohol over the last 30 days. Similarly, 44.4% (n = 12) of the respondents had smoked cigarettes and 15.5% (n = 9) of the respondents smoked shisha in the past 30 days. Furthermore, about 28.6% (n = 12) of the participants reported the use of Ganja in the past month. Table 2.

**Table 2:** Substance use, risky sexual behavior, and negative life eventsamongJimma university main campus regular students, April 2016. (N = 556)

Variables	Number (n)	Percentage (%)
Alcohol use		
Lifetime		
No	345	62.1
Yes	211	37.9
Current		
No	160	75.8
Yes	51	24.2
Khat use		
Lifetime		
No	438	78.8
Yes	118	21.2
Current		
No	40	34
Yes	78	66
Cigarette smoking		
Lifetime		
No	529	95.1
Yes	27	4.9
Current		
No	15	55.6
Yes	12	44.4
Shisha use		
Lifetime		
No	498	89.6
Yes	58	10.4
Current		
No	49	84.5
Yes	9	15.5
Ganja use		
Lifetime		
No	514	92.4
Yes	42	7.6
Current		
No	30	71.4
Yes	12	28.6
Multiple sexual partners		
No	60	10.8
Yes	113	20.3
Sex after drinking		
No	516	92.8
Yes	40	7.2
Being hit by a sexual partner		
No	509	91.5
Yes	47	8.5
Forced to have sex		
No	500	89.9
Yes	56	10.1
Childhood physical abuse		
No	377	67.8
Yes	179	32.2
Childhood sexual abuse		
No	518	93.2
Yes	38	6.8
Childhood emotional abuse		
No	494	88.8
Yes	62	11.2
Childhood neglect		
No	493	88.7
Yes	63	11.3
Witnessing parental violence		
No	372	66.9
Yes	184	33.1
Death of parents before the age of 11		
No	16	2.8
Yes	65	11.7
Multiple caretakers in early life		
No	53	9.5
Yes	26	4.7

### Prevalence of depression

Nearly one-third 28.2%, (n = 157) of the participants had depression. A total of 40.6% (n = 226) of the participants were free from depression (Normal), 31.1% (n = 173) had borderline clinical depression, 14.4% (n = 80) had mild depression, 9.9% (n = 55) had moderate depression and 4% (n = 22) had severe depression.

### Factors associated with depression

Bivariate logistic regression analysis showed, being single, having a poor parental relationship, chronic physical illness, family history of mental illness, having sex after drinking, being hit by a sexual partner, being forced to have sex, having childhood physical, emotional and sexual abuse, witnessing parental violence, earning 500–999ETB pocket money and academically promoted status were associated with depression. Multicollinearity and Lemeshow-Hosmer test of model fitness tests were done before the final model. Finally, multivariate logistic regression analysis revealed a family history of mental illness, college type, being hit by a sexual partner; childhood emotional abuse, academic performance, pocket money, and sex after drinking had a significant association with depression.

Accordingly, the odds of having depression were nearly two and a half fold higher (OR = 2.307, 95% CI 1.055–5.049) among students who had a family member with mental illness as compared to their counterparts. Similarly, the odds of having depression were nearly two and a half fold higher (OR = 2.582, 95% CI 1.332–5.008) among students who were from the college of Social science and humanity than students from the college of law and governance. Additionally, the odds of having depression were approximately four times more likely (OR = 3.722, 95% CI 1.818–7.619) in the students who had sex after drinking alcohol than their counterparts. Students who have been hit by sexual partners were three times (OR = 3.132, 95%CI 1.561–6.283) more likely to develop depression than students who had no such events. Likewise, students who reported childhood emotional abuse were two times more likely (OR = 2.167, 95%CI 1.169–4.017) to report depression than their counterparts. Furthermore, students who have reported getting monthly pocket money between 500 and 999(ETB) had a 55% low risk of having depression (OR = 0.450, 95%CI 0.204–0.995) than students with pocket money greater than 1000(ETB). Finally, the odds of having depression among students with promoted academic status were three-fold higher than students who have passed with great distinction academic status (OR = 2.912, 95%CI 1.063–7.975). Table 3.

**Table 3:** Multivariate analysis of factors associated with depression among participants at Jimma university main campus regular student, April 2016. (N = 556)

Variables	Depression			AOR & 95% CI	P-value
	No N (%)	Yes N (%)			
Family members with mental illness					
No	378 (72.8)	141 (27.2)	1	1	1
Yes	21 (56.8)	16 (43.2)	2.043 (1.036–4.026)*	2.307 (1.055–5.049)	0.036**
College					
CHS	182 (75.8)	58 (24.2)	1.243 (0.701–2.205)	1.273 (0.682–2.377)	0.448
CSSH	71 (56.8)	54 (43.2)	2.966 (1.619–5.434)*	2.582 (1.332–5.008)	0.005**
CNCS	68 (73.1)	25 (26.9)	1.434 (0.732–2.807)	1.523 (0.731–3.173)	0.261
CLG	78 (79.6)	20 (20.4)	1	1	1
Sex after drinking					
No	380 (73.6)	136 (26.4)	1	1	1
Yes	19 (47.5)	21 (52.5)	3.088 (1.611–5.920)*	3.722(1.818–7.619)	< 0.001**
Being hit by a sexual partner					
No	381 (74.9)	128 (25.1)	1	1	1
Yes	18 (38.3)	29 (61.7)	4.796 (2.576–8.926)*	3.132 (1.561–6.283)	0.001**
Childhood emotional abuse					
No	365 (73.9)	129 (26.1)	1	1	1
Yes	34 (54.8)	28 (45.2)	2.330 (1.359–3.994)*	2.167 (1.169–4.017)	0.014**
Pocket money(Ethiopian Birr)					
< 100	15 (78.9)	4 (21.1)	0.351 (0.100–1.229)	0.348 (0.088–1.368)	0.131
100–299	55 (62.5)	33 (37.5)	0.789 (0.378–1.649)	0.905 (0.386–2.118)	0.817
300–499	161 (73.5)	58 (26.5)	0.474 (0.243–0.924)*	0.489 (0.224–1.071)	0.074
500–999	143 (76.9)	43 (23.1)	0.396 (0.199–0.787)*	0.450 (0.204–0.995)	0.049**
> 1000	25 (56.8)	19 (43.2)	1	1	1
Academic performance					
Warned	3 (75)	1 (25)	2.267 (0.196–26.271)	1.812 (0.133–24.738)	0.656
Promoted	228 (66.1)	117 (33.9)	3.489 (1.330–9.158)*	2.912 (1.063–7.975)	0.038**
Distinction	134 (80.2)	33 (19.8)	1.675 (0.608–4.612)	1.327 (0.462–3.812)	0.599
Great distinction	34 (87.2)	5 (12.8)	1	1	1

*CHS* College of health science, *CSSH* College of social science and Humanity, *CNCS*College of natural and computational science, *CLG* College of law and Governance

*Variables with p ≤ 0.25 on Binary logistic regression

**Variables with p < 0.05 on multiple logistic regression or have a significant association

## Discussion

The finding of this study showed that the prevalence of depression among Jimma university regular undergraduate students was 28.2% ± 3.74 (95% CI 24.46%–31.94%). This figure is largely falling within the prevalence rates reported across different studies in a similar study population. The finding was similar to the studies carried out in Addis Ababa 27.7% [62], Hawassa 30% [63], and Ambo 32.2% [42] university students. But the current study finding was higher than the study done in Adama 21.6% University [64]. The difference could be explained by the Adama study was used a lower sample size (413) and different assessment tools (SRQ-20). However, this study finding was lower than the study done in Jimma 58.4% [65] and Addis Ababa 51.3% [66]. The variation might be due to the difference in the data collection tool in which previous studies were used 10-item Kessler Psychological Distress Scale and Hospital anxiety and depression scale (HADS). The other reason might be the difference in study participant’s sociodemographic and economic characteristics like age, marital status, address, educational status, parent educational level, and family monthly income. In this study, the majority of the study participant’s parents don’t have advanced educational status, this may expose students with low parental educational status to have lower self-esteem and more pressure on their psychology [53, 67]. Additionally, lack of free and open discussion about various stressful issues in the University, and sharing university life experiences might lead those students from low parental education status more vulnerable to depression [68]. Another difference could be the family’s monthly income. In this study the majority of the study respondent’s families had better income, this may lead students with better family monthly income less worried about the academic achievements, more indulged in substances, and risky practices, this may ultimately contribute to the prevalence of depression in the study setting.

In this study 31.1% of the respondents had borderline depression, 14.4% of the participants had mild depression, 9.9% had moderate depression and 4% had severe depression. A consistent finding was reported from the study done among Indian University students, that disclosed 37.7%, 13.1%, and 2.4% of the students were suffering from moderate, severe, and extremely severe depression [69], however this study finding was different from the study done in China, where 40.1% of the students were classified as minor depression (borderline), 8.4% as moderate and 3.3% as the severe depression [20]. The reason for the difference could be different cutoff points used for depression symptoms severity categorization between the two studies.

The results of this study showed that having a family history of mental illness is a significant predictor of depression. This is in line with the study done in India medical students, Newzeland, and Germany which showed an individual from family members with mental illness are more prone to develop depression [14, 70, 71]. This might be explained by the fact that mental illness has a genetic base, families are stigmatized and there is a lot of burden on the family members regarding financial expenses and giving care for the patient as well as the offsprings might be stressed and worried about their parent’s health condition, this might increase the risk of having depression [1, 2].

This study also revealed that students from the college of social science and humanity had a higher depression rate than students from the college of law and governance. Consistent findings were reported from the study done in Egypt and India [13, 69]. Although the reasons are not clear at the moment, it may be related to better job prospects for law graduates in the Ethiopian context. This issue requires the attention of the academic administration of the Humanities and Social Science departments of the university, hence special classes should be organized for the Humanities and Social Science students to make them psychologically more competent to develop skills and adopt effective strategies to manage stress and symptoms of depression. Another reason could be, in the current study students from the College of social science and humanity were reported more alcohol and other substance use, which might lead them to develop depression compared to students from the College of Law. Further qualitative and quantitative studies needed to elucidate this finding.

Risky sexual behavior like having sex after drinking was associated with depression in this study. Similar findings were reported from the studies done in Kenya [39] and Jimma University [72]. This might be explained by the fact that students who are abusing substances are more prone to develop depression. Moreover, participating in a risky sexual activity may prone the students to have guilt feeling and worry about acquiring STI including HIV/AIDS, which might lead to psychological distress mainly depression.

In the current study history of hit by a sexual partner and childhood emotional abuse were significant association with depression. Similar results were found from the studies conducted among Kenya university students and the USA [39, 73]. This might be explained by people expect care and love from a sexual partner when this is not met they may be disappointed and dissatisfied which might lead them to have divorce and abuse substances [74]. Besides, child abuse might lead the individual to develop short term and longterm psychological damage and adopt behavioral risk factors such as smoking, alcohol abuse, poor diet, and lack of exercise which in turn lead to depression [75]. Moreover, emotionally abused children are more likely to be withdrawn and had a poor interaction with the family as well as the community this increases the risk of having depression in these groups [76].

In this study, academic performance and depression were found to have a strong association. Students who passed with promoted academic status were more prone to develop depression than students who passed with great distinction. A consistent findings were reported from the studies conducted in Saudi Arabia and Pakistan [13, 77]. This could be explained by depression and poor academic performance has a bi-directional association. Various studies found that depression deteriorates cognitive functioning like memory, attention, concentration, abstract reasoning, and judgment which trigger the students to achieve poor academic activities. in another pole students with poor academic status will be tensioned, worried, and end up with substances for improving their concentration, which can ultimately lead them to have depression [26, 78].

According to this study finding, depression is associated with monthly pocket money. Students who got low pocket money were less likely to develop depression compared to the students who received higher pocket money. It is known that most of the literature reports that having financial problems (struggles) leads students to develop stress and depression however this study finding is inconsistent with the study report from Egypt [38]. In Ethiopia University tuition fee(educational, dormitory and meal) will be paid after the students graduated and got Job, it is also well known that the majority of the students join from rural and poor families so, universities will be a better comfortable setting for many students, these students can get a free meal, better accommodation than home, this will make those students with low pocket money less stressed to have financial problems. But in opposite students with more monthly pocket money come from urban areas where substance use is more common compared to students from the rural area. Another reason could be students who get more monthly pocket money are less cared for in their academic achievements and enjoying the money, this commonly ends up with warning academic status, this might make the students stressed, abuse substance, hopeless, and could be a risk factor for depression. Further researches needed to elucidate the reasons.

## Conclusion

This study has shown a high prevalence of depression among Jimma university students. Additionally, the study has revealed statistically significant association between depression and academic, socio-economic, abuse, substance use, risky sexual behavior and family mental health problem variables. Based on the findings, it is better if the Ethiopian Ministry of Education and Health work in collaboration with higher administrative bodies of the University to design mental health promotion and prevention strategies for the prevention of the new incidences of depression and to provide appropriate mental health treatment services for the affected students. Further longitudinal and qualitative studies are needed to understand the cause-effect relationship between the outome and associated variables.

## Data Availability

The datasets used and/or analyzed during the current study are available from the corresponding author on reasonable request.
